# Interrelated ecological impacts of climate change on an apex predator

**DOI:** 10.1002/eap.2071

**Published:** 2020-02-04

**Authors:** Kristin L. Laidre, Stephen Atkinson, Eric V. Regehr, Harry L. Stern, Erik W. Born, Øystein Wiig, Nicholas J. Lunn, Markus Dyck

**Affiliations:** ^1^ Polar Science Center Applied Physics Laboratory University of Washington Seattle Washington 98105 USA; ^2^ Wildlife Research Section Department of Environment Government of Nunavut P.O. Box 209 Igloolik Nunavut X0A 0L0 Canada; ^3^ Greenland Institute of Natural Resources P.O. Box 570 3900 Nuuk Greenland; ^4^ Natural History Museum University of Oslo P.O. Box 1172 Blindern N‐0318 Oslo Norway; ^5^ Environment and Climate Change Canada CW‐422 Biological Sciences Building University of Alberta Edmonton Alberta T6G 2E9 Canada

**Keywords:** Arctic, body condition, climate change, genetic mark–recapture, Greenland, Nunavut, polar bear, reproduction, sea ice

## Abstract

Climate change has broad ecological implications for species that rely on sensitive habitats. For some top predators, loss of habitat is expected to lead to cascading behavioral, nutritional, and reproductive changes that ultimately accelerate population declines. In the case of the polar bear (*Ursus maritimus*), declining Arctic sea ice reduces access to prey and lengthens seasonal fasting periods. We used a novel combination of physical capture, biopsy darting, and visual aerial observation data to project reproductive performance for polar bears by linking sea ice loss to changes in habitat use, body condition (i.e., fatness), and cub production. Satellite telemetry data from 43 (1991–1997) and 38 (2009–2015) adult female polar bears in the Baffin Bay subpopulation showed that bears now spend an additional 30 d on land (90 d in total) in the 2000s compared to the 1990s, a change closely correlated with changes in spring sea ice breakup and fall sea ice formation. Body condition declined for all sex, age, and reproductive classes and was positively correlated with sea ice availability in the current and previous year. Furthermore, cub litter size was positively correlated with maternal condition and spring breakup date (i.e., later breakup leading to larger litters), and negatively correlated with the duration of the ice‐free period (i.e., longer ice‐free periods leading to smaller litters). Based on these relationships, we projected reproductive performance three polar bear generations into the future (approximately 35 yr). Results indicate that two‐cub litters, previously the norm, could largely disappear from Baffin Bay as sea ice loss continues. Our findings demonstrate how concurrent analysis of multiple data types collected over long periods from polar bears can provide a mechanistic understanding of the ecological implications of climate change. This information is needed for long‐term conservation planning, which includes quantitative harvest risk assessments that incorporate estimated or assumed trends in future environmental carrying capacity.

## Introduction

Progressive changes in the abiotic environment are expected to result in profound ecological disruptions for Arctic fauna, particularly sea‐ice‐dependent species (Post et al. 2009, Laidre et al. 2015a, Reimer et al. 2019). Even if greenhouse gases, the primary drivers of climate change, were limited immediately, sea ice loss would continue for several decades because of inertia in the climate system (Overland and Wang 2013). Therefore, continued unprecedented changes in Arctic marine mammal habitats are inevitable and can be expected to result in unprecedented challenges for conservation and resource managers (Derocher et al. 2013, Regehr et al. 2017a).

Polar bears depend on sea ice for nearly all aspects of their life history and are distributed throughout ice‐covered areas of the circumpolar Arctic in 19 subpopulations (PBSG 2018). They occur in highest densities on annual ice over the continental shelf and within the channels of archipelagos (Laidre et al. 2015a). Polar bears require sea ice as a platform for hunting ringed seals (*Phoca hispida*) and bearded seals (*Erignathus barbatus*; Stirling et al. 1999). Sea ice also facilitates extensive seasonal movements, mating activities and, in some areas, maternal denning (PBSG 2018). As a consequence, the loss of sea ice has the potential to disrupt multiple aspects of the polar bear life cycle (Stern and Laidre 2016). Earlier sea ice breakup (i.e., melting in the spring) or reductions in optimal ice habitat result in reductions in body condition, survival, reproduction, and abundance (Stirling et al. 1999, Regehr et al. 2007, 2010, Bromaghin et al. 2015). This also leads to nutritionally stressed polar bears appearing more often on land, resulting in increased human–bear conflicts (Wilder et al. 2017). Variability in the current status of the world's polar bear subpopulations emphasizes the need for predictive tools developed on a regional scale using empirical field data specific to subpopulations (PBSG 2018).

For many populations of large, long‐lived mammals, density‐dependent regulation (e.g., due to increasing abundance or declining environmental carrying capacity) negatively affects body condition and reproduction prior to adult survival (Fowler 1987, 1990, Zedrosser et al. 2006). As such, indices of body condition and reproduction are used to monitor polar bears across their circumpolar range (Stirling and Derocher 2012, Vongraven et al. 2012). For several subpopulations with long‐term capture–recapture data, declines in these indices associated with sea ice loss due to climate warming (Stirling et al. 1999, Rode et al. 2010, Obbard et al. 2016) have been detected prior to declines in survival and abundance (Regehr et al. 2007, 2010, Lunn et al. 2016, Obbard et al. 2018). Because these metrics are often easier to obtain than precise estimates of survival, they can be used to track long‐term trends in subpopulation status, parameterize population viability models, and inform risk assessments (Regehr et al. 2017b).

The annual life cycle of polar bears is characterized by large seasonal changes in body condition (Atkinson and Ramsay 1995). In general, body condition improves during the spring and early summer when juvenile seals are abundant and vulnerable to predation (Smith 1980, Stirling and Øritsland 1995). This period of hyperphagia is followed by a period of food scarcity in the late summer and fall when sea ice reaches a minimum throughout the Arctic (Serreze et al. 2007). During this season, bears in some regions are forced onto land where access to seals and other marine mammal prey is greatly reduced (e.g., Derocher and Stirling 1990, Ferguson et al. 2000a, Stirling and Parkinson 2006, Laidre et al. 2018a, b). In other regions, bears may remain on offshore pack ice (e.g., Wiig et al. 2003, Laidre et al. 2015b) where they likely face reduced access to seals over deep and less‐productive waters of the polar basin (Amstrup et al. 2000, Stirling 2002, Atwood et al. 2016). During this period of food scarcity, bears rely on nutrients and energy stored within adipose and other body tissues to meet a significant portion of their metabolic requirements. Consequently, body condition generally declines until sea ice reforms in the late fall and early winter, allowing polar bears to resume on‐ice foraging (Atkinson and Ramsay 1995, Derocher and Stirling 1995, Atkinson et al. 1996, Rode et al. 2012, Obbard et al. 2016). Additionally, pregnant females enter dens in the fall where they rely exclusively on body stores to support gestation and early‐ to mid‐lactation over a period of 6–8 months (Atkinson and Ramsay 1995, Derocher and Stirling 1995).

For the subpopulations where bears are forced onto land, ice loss results in longer periods of fasting with physical and reproductive consequences (Stirling et al. 1999, Regehr et al. 2007, Molnár et al. 2010, 2014, Whiteman et al. 2017). Polar bears in the Baffin Bay (BB) subpopulation spend the summer onshore when Baffin Bay is ice free (Ferguson et al. 1997, 2000a). Sea ice declines have been documented in Baffin Bay (Stern and Laidre 2016, Laidre et al. 2018a, b), yet the potential effects on polar bear behavior, body condition, and reproduction have received little attention. Here, we use three independent and concurrent data types: satellite telemetry data, and visual observations on body condition and reproduction from both physical captures and remote biopsies, referenced to the same time periods and locations to assess changes in time spent on shore, body condition, and reproductive performance over nearly three decades. We use model output to predict reproductive performance of BB polar bears three generations into the future under assumptions of continued and predicted large‐scale sea ice loss. We also present the first analyses for polar bears using reproductive and body condition metrics obtained from visual surveys, which can be used as a monitoring tool between periods of more in‐depth demographic studies.

## Methods

### Study area

**Figure 1:**
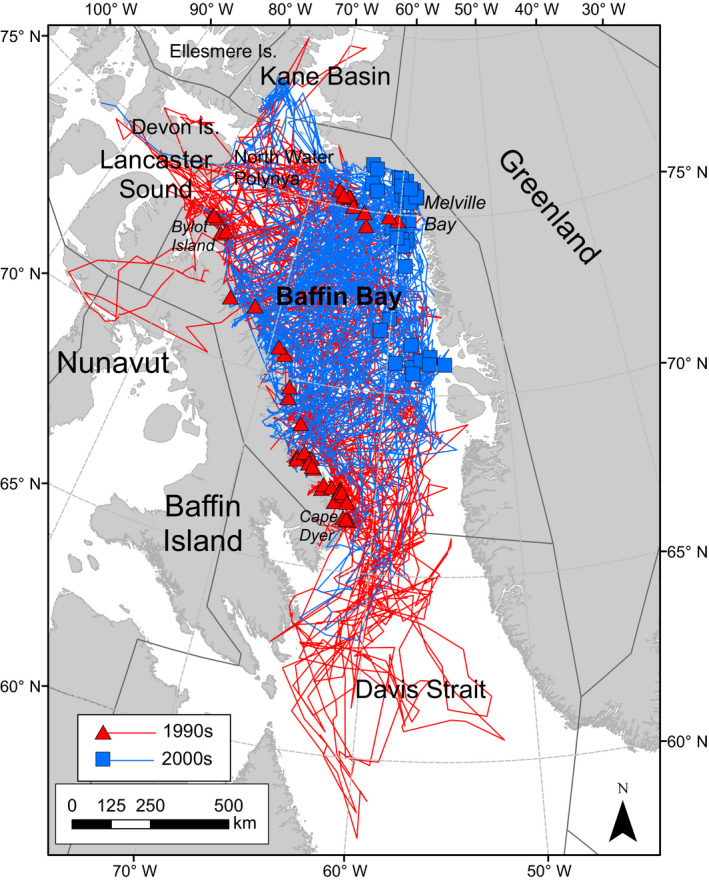
Capture locations and movements of adult female polar bears in Baffin Bay shown in red for the 1990s and blue for the 2000s. PBSG (2018) subpopulation boundaries are shown on the map with black lines.

Baffin Bay is bounded by Greenland to the east, Baffin Island to the west, the North Water polynya to the north, and Davis Strait to the south (Fig. 1). The boundaries of the BB polar bear subpopulation encompass ~1 million km^2^ (marine area 656,000 km^2^) covering portions of Baffin Island and all of Bylot Island (66.2° N to 73.8° N) in Nunavut, Canada, as well as parts of West and Northwest Greenland (66.0° N to 77.0° N; Taylor et al. 2005). During late spring and summer, sea ice melt begins along the southwest coast of Greenland and proceeds north and west across Baffin Bay. The last remnants of ice typically occur off the coast of Baffin Island, although, in some years, patches of ice may remain in Melville Bay in northwest Greenland throughout the summer (Taylor et al. 2001; this study).

### Sea ice data

Methods for sea ice analyses are described in detail in Stern and Laidre (2016) and briefly reviewed here. We used daily gridded sea ice concentration data from satellites for the period 1979–2018 (Cavalieri et al. 1996; updated yearly) to calculate the sea ice area in Baffin Bay, which is typically 100% ice covered in March and 0% ice covered in September. For each year, we determined the date in spring when the ice coverage dropped below 50% and the date in fall when the ice coverage rose above 50%. These metrics, referred to as spring and fall transition dates, respectively, were used in analyses of polar bear body condition and reproduction. All sea ice metrics were calculated relative to the area of ocean within the BB subpopulation boundary with depth <300 m because shallow waters of the continental shelf constitute the most important habitats for polar bears (e.g., Laidre et al. 2018b).

### Satellite telemetry location data from polar bears

Between mid‐March and mid‐April 2009–2013, we used an Ecureuil AS350 helicopter to search for polar bears on the fast and pack ice of Baffin Bay in Northwest Greenland. Searches occurred to a distance of ~150 km from the coast, and along the coast including areas with consolidated glacier ice at the foot of marine‐terminating glaciers in Melville Bay. Polar bears were temporarily sedated using standard chemical immobilization techniques (Stirling et al. 1989). Reproductive status and field estimates of age were recorded. Age was determined based on previous capture history, known birth year (in the case of COY and YRL), or estimated based on counts of annular rings in an extracted vestigial premolar tooth (Calvert and Ramsay 1998). Adult females (AF) were defined as ≥5 yr old (Rosing‐Asvid et al. 2002), and classified as independent, with an adult male (AM, ≥6 yr old) for breeding activity, or with dependent offspring consisting of cubs‐of‐the‐year (COY), yearlings (YRL), or 2‐yr olds (2YR; Table 1). Adult female polar bears were fitted with TAW‐4610H satellite radio collars (Telonics, Mesa, Arizona, USA) that provided information on geographic location, internal transmitter temperature, and activity. Collars were programmed to transmit during one 6‐h period each day on 4‐d intervals.

**Table 1:** Number of individual adult female (AF) polar bears in the Baffin Bay subpopulation tracked with satellite collars in the 1990s and 2000s

Time span	AF alone	AF+AM	AF+COY	AF+YRL	AF + 2YR	Sum
1990s	9	0	19	13	2	43
2000s	10	2	6	12	8	38

*Note*

2YR, 2‐yr‐old cub; AF, adult female; AM, adult male; COY, cub‐of‐the‐year; YRL, 1‐yr‐old cub.

We combined movement data from these bears with historical data from 43 adult female polar bears from BB that had been collared as part of subpopulation studies conducted during 1991–1997 (Ferguson et al. 2000b, Taylor et al. 2001) to evaluate temporal changes in the date bears arrived on land in summer and the date they returned to the sea ice in fall.

Data on location from all polar bears were collected via the Argos Location Service Plus system (Toulouse, France). Location qualities were assigned by ARGOS to each position, with qualities of 0–3 estimated to have errors of 1.5 km or less, whereas positions categorized as “A,” “B,” or “Z” did not have a predicted accuracy (Argos 2016). Unrealistic and poor‐quality locations were removed using a speed and angle filter in R version 3.5.2 (R Development Core Team 2016) using the package argosfilter (Freitas et al. 2008). The filtering algorithm removed locations that exceeded a maximum between‐location speed of 10 km/h (based on previous movement studies of polar bears; Laidre et al. 2013) and angle (measured from the track between three successive locations; set to the default). The resulting locations for each bear were reduced to a single position per day to reduce autocorrelation, standardize temporal sampling, and reduce the effects of variable duty cycling among collars. To obtain a daily position for each collar, the first, highest‐quality location within the period of peak satellite passage was selected (generally around 12:00 GMT each day). The resulting daily positions only consisted of ARGOS location qualities 1–3. Distances between successive daily positions were calculated as the great circle route and used to compute minimum daily displacements.

Due to varying study objectives in the two time periods, different duty cycles were used to extend battery life or gather information from specific time periods. The 1990s collars were programmed to transmit on intervals ranging from 1 to 6 d, while the 2000s collars all transmitted on 4‐d intervals. We sub‐sampled the 1990s data and created a strict 4‐, 5‐ or 6‐ d interval time series for each individual to best match the 2000s data. This ensured that the impact of serial autocorrelation was largely consistent between study periods.

Polar bears collared in this study ranged over the entire Baffin Bay region (Laidre et al. 2018a, b). All captures occurred within the BB subpopulation boundaries (PBSG 2018; Fig. 1). However, seasonal and geographic differences in capture locations occurred between the two periods of field work. In the 1990s, it was possible to deploy collars on AFs on both the Canadian and Greenlandic sides of Baffin Bay (Fig. 1). However, in the 2000s, it was only possible to deploy collars on the Greenlandic side (between approximately 72°–76° N) due to opposition to immobilization and handling of polar bears in Nunavut, Canada (SWG 2016, Laidre et al. 2018a). Prior to conducting inter‐decadal analyses of polar bear movements, we evaluated the 1990s and 2000s data to identify potential effects of differences in collar deployment location. This included comparing fall area use and resource–selection models for habitat use (Laidre et al. 2018a, b). These analyses demonstrated that bears captured in West Greenland in spring used the same geographical areas, had the same seasonal movement patterns, and the same habitat preferences as bears captured on the east coast of Baffin Island in fall, thus providing a solid basis for comparing telemetry data from the two study periods (see Laidre et al. 2018a, b for details).

### Arrival and departure dates on land

We used satellite telemetry locations to compare the phenology of land use by polar bears in Baffin Bay between the two time periods 1991–1997 and 2009–2015. We excluded the few resident bears that remained in Melville Bay (West Greenland) for the entire tracking period as their use of glacial ice and land throughout the summer could not be differentiated. We quantified the date individual AF polar bears arrived on Baffin Island in summer, the duration of time spent on land, and the date they returned to the sea ice in fall. In some cases, individual bears contributed more than one arrival date because they were tracked over multiple years; these were considered to be independent because land use by polar bears is primarily a function of sea ice availability (e.g., Atwood et al. 2016). Including an individual random effect in the comparison of land use across decades did not affect the results.

We considered a bear to be on land if its Argos location was within 5 km of the high‐resolution coastline as identified by the zero elevation contour of the International Bathymetric Chart of the Arctic Ocean (IBCAO) digital elevation model (Jakobsson et al. 2012). This 5‐km buffer was used to encompass small barrier islands that may be used by polar bears in the summer but may not be depicted as land, and to account for low accuracy of locations. Bears were required to enter and remain within the 5‐km buffer for at least 14 d before they were considered to be “on land.” A similar criterion was used for bears departing land (≥14 d on the ice). For all AFs identified to be in maternity dens (Escajeda et al. 2018), we excluded dates of return to the sea ice in spring, as the maternity denning period dictated the date of return, not the formation of the sea ice. For pairs of positions that were separated by 4 or 8 d, we linearly interpolated the date of arrival on land or departure from land and compared decadal means with a two‐sided *t* test. We excluded data when observed locations were separated by >8 d, except when bears were offshore in summer in open water (<15% sea ice concentration) and the subsequent position was on land. In these cases, there were data gaps (12–30 d) in locations due to the inability of satellite collars to transmit data when polar bears were swimming (Pagano et al. 2012) from central Baffin Bay to the shore.

### Body condition indices of polar bears on land

We evaluated body condition and reproduction using data from bears sampled for mark–recapture studies on eastern Baffin and Bylot islands, from late August to mid‐October, in 1993–1995, 1997, and 2011–2013. During the 1990s, bears were sampled by physical capture (Taylor et al. 2005) during which sex and reproductive class were recorded. Capture date, location, and group size (i.e., for family groups or bachelor groups consisting of unrelated males) were also recorded. During the 2000s, bears were sampled from the air with remote biopsy darting and subsequent genetic analysis to determine sex (SWG 2016). We estimated age class (COY, YRL, subadult [2–4 yr old], and adult) by visual observation at 3–6 m altitude. Observers used multiple cues, including the size of an individual relative to its surroundings or accompanying bears, membership in a family group (i.e., mothers with COY or YRL, which could be visually aged without error), secondary sexual characteristics (e.g., foreleg guard hairs typical of adult males), body shape and proportions, presence of scars (most often seen on adult males), and observations of urination (i.e., urine dribbling from under tail in females). Field notes assisted in post‐hoc reassessment of sex and age class once genetic sex was determined from tissue samples. Also, age class was later verified for some bears from previous or future observations in which an individual was captured and physically examined or was matched via DNA to membership in a known family group. Because a larger geographic area was sampled in the 2000s, we standardized observations by only including data from both decades collected within the same sampling area.

During both sampling periods, all bears were assigned a fatness index (FI) score from 1 to 5, where 1 and 5 represent the leanest and fattest bears, respectively (Stirling et al. 2008). During the 1990s, FI scores were based on physical examination (e.g., palpation of fat along hips and spine). During the 2000s, FI scores were based on aerial observation by one of three experienced observers, with 79% of observations made by one observer (S. Atkinson). Due to the low frequencies of bears scored as 1 or 5, FI scores were simplified to a three‐point body condition score (BCS) of poor (BCS = 1; FI = 1 or 2), fair (BCS = 2; FI = 3) and good (BCS = 3; FI = 4 or 5). Similar recategorization of FI scores has been recommended or used in other polar bear studies (Stirling et al. 2008, Vongraven et al. 2012).

We analyzed patterns in body condition using multinomial logistic regression with BCS for individual *i* in year *t* considered as a three‐level response variable (i.e., BCS_*it*_ = 1, 2, or 3). Repeat observations of individual *i* in the same year were excluded. Observations of individual *i* in different years were retained, following the example of Stirling et al. (2008), because body condition in polar bears fluctuates widely within and among years as a function of hunting success and environmental conditions (Atkinson and Ramsay 1995, Atkinson et al. 1996). We modeled BCS as a function of categorical and continuous predictors reflecting biological hypotheses for the effects of sex, age, and reproductive class, sea ice conditions, within‐year timing of the observation, and decade (i.e., 1990s vs 2000s; Rode et al. 2010, 2012). Body condition scores of dependent young were excluded from analyses because these animals were dependent on their mother.

Analyses followed a three‐step process. First, we created the general model: BCS_*it*_ = β_0_ + β_1_ AFwC_*it*_ + β_2_ SUB_*it*_ + β_3_ AM_*it*_ + β_4_ p2000_*t*_ + β_5_ springtran_*t*_ + β_6_ ts.springtran_*it*_ + β_7_ durfree_*t*−1_ + β_8_ AFwC_*it*_ × springtran_*t*_ + β_9_ AFwC_*it*_ × durfree_*t*−1_ + β_10_ SUB_*it*_ × springtran_*t*_ + β_11_ SUB_*it*_ × durfree_*t*−1_ + β_12_ AM_*it*_ × springtran_*t*_ + β_13_ AM_*it*_ × durfree_*t*−1_, where AFwC, SUB, and AM are individual covariates designating adult females with dependent young, subadults, and adult males, respectively; springtran_*t*_ is the spring sea ice transition date in year *t*; ts.springtran_*it*_ is the number of days elapsed between the spring transition date and the observation of bear *i* in year *t*; durfree_*t*−1_ is the duration of the ice‐free season, defined as the number of days elapsed between the spring and autumn transitions dates, in year *t* − 1; and p2000_*t*_ is a binary indicator designating observations in the 2000s. We hypothesized that increased availability of sea ice in year *t* and *t* − 1 (i.e., larger values of springtran_*t*_ and smaller values of durfree_*t*−1_) would be positively correlated with BCS (Obbard et al. 2016). Interaction terms were included due to variation in nutritional requirements across sex, age, and reproductive classes (Rode et al. 2014), and the potential for environmental conditions to affect these classes differently (Regehr et al. 2017a). We hypothesized that single bears (i.e., AMs and AFs without dependent young) would have the highest BCS, and that BCS for subadult bears would be most sensitive to environmental fluctuations. To ensure the general model was a suitable starting point for model selection, we checked for collinear predictors and evaluated goodness‐of‐fit (GOF) using Hosmer and Lemeshow tests (Hosmer et al. 2013). The second step of the analytical process was to develop a candidate model set representing all combinations of main effects and interaction terms in the general model, with a marginality constraint to ensure that interactions were only included if the corresponding main effects were included. In the third step, we performed model selection using Akaike's information criterion (AIC) and derived model‐averaged parameters for all models with ΔAIC < 4 (Burnham and Anderson 2002). Modeling was performed in the R programming language version 3.5.2 (R Development Core Team 2016) using package nnet (Venables and Ripley 2002) for multinomial logistic regression and package MuMIn (Barton 2018) for multimodel inference.

### Reproductive indices of polar bears on land

We evaluated reproductive metrics that have been identified as important for monitoring polar bears (Vongraven et al. 2012). We modeled litter size as a function of individual and environmental covariates using logistic regression. Although triplet litters may occur in some productive subpopulations (e.g., Regehr et al. 2018), only 2 of 299 litters for the BB subpopulation consisted of three cubs. Thus, we considered litter size for adult female *i* in year *t* to be a binary response variable (i.e., ls_*it*_ = 1 or 2). Analyses for COY and YRL litters were performed separately, following the same three‐step modeling approach employed for body condition. The general model for COY was ls_*it*_ = β_0_ + β_1_ springtran_*t*_ + β_2_ ts.springtran_*it*_ + β_3_ durfree_*t*−1_ + β_4_ p2000_*t*_ + β_5_ BCS_*it*_, where BCS_*it*_ is a three‐level factor for body condition of a cub's mother at the time of sampling. The general model for YRL was ls_*it*_ = β_0_ + β_1_ ts.springtran_*it*_ + β_2_ durfree_*t*−1_ + β_3_ durfree_*t*−2_ + β_4_ p2000_*t*_ + β_5_ BCS_*it*_, where durfree_*t*−2_ is the duration of the ice‐free period in year *t* − 2, which we hypothesized was related to maternal body condition and thus reproductive success in the year that the litter was produced (Derocher and Stirling 1995). We did not include springtran_*t*_ in this model because it had a correlation coefficient of −0.95 with durfree_*t* − 2_.

After evaluating patterns in litter size, we calculated the mean number of dependent young (COY or YRL) per adult female in each year and evaluated changes between the 1990s and 2000s. These metrics have been used as indices of productivity for other polar bear subpopulations (e.g., Stirling et al. 1980, Derocher and Stirling 1995, Rode et al. 2010, Peacock et al. 2013, Regehr et al. 2015). Uncertainty was quantified using a bootstrap procedure with 10,000 iterations, during which observations of individual polar bears were resampled with replacement and reproductive metrics were calculated from the resampled data. We did not evaluate reproductive metrics for 2‐yr‐old bears because most of them had been weaned by autumn and thus were classified as subadults.

### Projecting reproductive metrics as a function of sea ice and body condition

We projected mean COY litter size using the most‐supported models for BCS and litter size, together with sea ice metrics projected forward in time with either a stable or declining trend. Uncertainty was quantified using a bootstrap procedure during which observations of individual polar bears were resampled with replacement, the most‐supported models were fit to the resampled data, stochastic predictions of BCS were generated based on multinomial probabilities from the BCS model together with projected sea ice conditions, and stochastic predictions of COY litter size were generated based on binomial probabilities from the litter size model together with stochastic values of BCS and projected sea ice conditions. Projections were performed for 35 yr into the future (approximately three polar bear generations; Regehr et al. 2016) using 1,000 bootstrap iterations.

## Results

### General patterns of summer movements between decades

Of the 38 AFs collared in BB in the 2000s, 7 bears (18.4%) remained on glaciers or on fast ice at glacier fronts in Melville Bay through summer, and in some cases for over 2 yr, and did not move to Baffin Island. No bears in the 1990s (*n* = 43) exhibited this behavior and all moved to Baffin Island for the ice‐free season. Bears that remained in West Greenland in the 2000s spent the entire on‐ice season on the fast ice near glacier fronts, and during the summer used glacial ice, land, or the glacier itself until the sea ice reformed.

### Sea ice melt patterns

**Figure 2:**
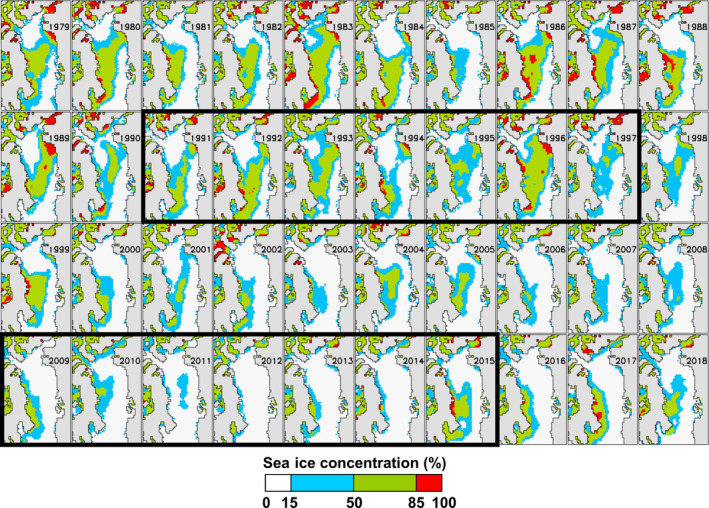
Sea ice concentration in Baffin Bay on 15 July of each year from 1979 (upper left) to 2018 (lower right). The two study periods (1991–1997 and 2009–2015) are indicated by black rectangles. In the early period, a sea ice bridge connected Baffin Island with northwest Greenland in six out of seven years. In the late period, the bridge had disappeared well before 15 July in every year.

Sea ice data indicate that the spatial pattern of melt in Baffin Bay has not changed over the last few decades, however the timing of that melt has shifted earlier in the season in the 2000s compared to the 1980s and 1990s (Fig. 2). Spring sea ice retreat in Baffin Bay begins with melting along the southwest coast of Greenland and progresses northward. At the same time, the North Water Polynya begins to melt out. During summer, these two open water areas connect as Melville Bay melts, severing the continuous ice bridge between Baffin Island and Greenland. The sea ice then continues to melt back toward the coast of Baffin Island. Occasionally a “sea ice island” offshore in central Baffin Bay becomes the last remnant of ice.

**Figure 3:**
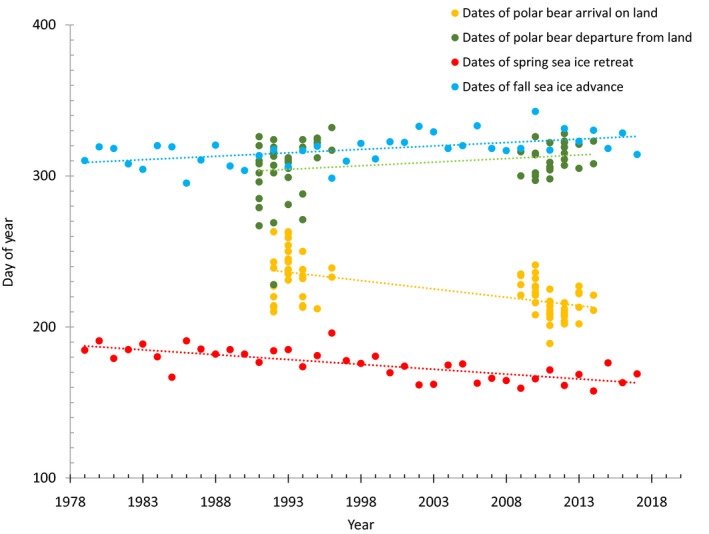
Dates of onshore arrival and departure of polar bears on Baffin Island relative to dates of sea ice fall advance and spring retreat.

Additionally, the sea ice bridge that was present between Melville Bay and Baffin Island in July through the 1990s was no longer available in the 2000s, essentially removing a surface substrate for travel across the bay for polar bears. The melt pattern up the coast of Greenland and across Melville Bay trended even earlier than the melt on the western side of Baffin Bay (Laidre et al. 2018a). In October and November, sea ice advanced southward through Baffin Bay, generally with the leading edge along the coast of Baffin Island. This pattern occurred later in the fall in the last decade than in the 1980s and 1990s (Fig. 3).

In Baffin Bay, sea ice retreated earlier in spring by 7 d/decade and advanced later in fall by 5 d/decade during 1979–2014, following trends in previous studies indicating loss of polar bear habitat (Stern and Laidre 2016, Laidre et al. 2018a, b, Fig. 3). The length of summer (number of days from retreat to advance) thus increased by 12 d/decade (see also Stern and Laidre 2016, SWG 2016).

### Arrival and departure dates on land

We determined onshore arrival dates for 78 AF polar bears in the 1990s and 2000s. Of these, 71 dates were for arrivals on Baffin Island, 5 on Devon Island, and 2 on Ellesmere Island. We also determined 71 departure dates in fall: 66 from Baffin Island, 3 from Devon Island, and 2 from Ellesmere Island. All arrivals and departures from Devon or Ellesmere islands occurred in the 1990s. We excluded the two dates from Ellesmere Island from summaries because it is located within multiyear sea ice of the Arctic Archipelago ecoregion, where break‐up dynamics are vastly different (Ferguson et al. 1997, 2000a).

The mean date of arrival on land in the 2000s (4 August, SD = 11 d, *n* = 46) was significantly earlier (by 20 d) than in the 1990s (24 August, SD = 16 d, *n* = 30; *P *<* *0.001). The mean date of departure from land in the 2000s (8 November, SD = 9 d, *n* = 27) was not statistically different than the mean date of departure in the 1990s (1 November, SD = 21 d, *n* = 42; *P *=* *0.07). The mean arrival latitude in the 2000s (70.3° N, SD = 1.3°, *n* = 46) was not statistically different than in the 1990s (70.7° N, SD = 3.0°, *n* = 30; *P *=* *0.50). Similarly, the mean departure latitude in the 2000s (70.4° N, SD = 1.4°, *n* = 27) was not statistically different than in the 1990s (69.7° N, SD = 3.0°, *n* = 42; *P *=* *0.20).

The decadal changes in the dates of AF arrival on land and departure from land are consistent with the long‐term changes in the sea ice metrics (Fig. 3). Polar bear arrival date exhibited a trend of −11 d/decade (SD = 1.6 d/decade, *P *<* *0.001) from 1992–1996 and 2009–2014, while the date of spring sea ice retreat had a long‐term trend of −6.4 d/decade (SD = 0.97 d/decade, *P *<* *0.001,) over the period 1979–2017. Polar bear departure date exhibited a trend of +4.7 d/decade (SD = 2.3 d/decade, *P *<* *0.05) from 1992–1996 and 2009–2014, while the date of fall sea ice advance had a long‐term trend of +4.6 d/decade (SD = 1.2 d/decade, *P *<* *0.001) over the period 1979–2017.

Duration of time spent on land was determined for 14 and 26 individuals in the 1990s and 2000s, respectively, for which both arrival and departure dates were available. The mean duration of time on land in the 2000s (95 d, SD = 16 d, range 56–120 d) was significantly longer (by 33 d) than in the 1990s (62 d, SD = 25 d, range 8–99 d; *P *<* *0.001). If only bears on Baffin Island were compared, the mean duration of time on land in the 2000s (95 d, SD = 16 d, range 56–120 d) was still significantly longer (by 20 d) than in the 1990s (75 d, SD = 14 d, range 54–99 d; *P *<* *0.001).

### Body condition

During the 1990s, BCS was assigned to 66 AFs without dependent young, 133 AFs with dependent young, 133 subadults, and 326 AMs. During the 2000s, BCS was assigned to 112 AFs without dependent young, 168 AFs with dependent young, 228 subadults, and 352 AMs (Table 2). When done by experienced observers, the system of visually assessing the sex and age class of polar bears from the air was accurate. Among a sample of captured or handled bears of known sex and age, observers correctly classified 97% (*n* = 62), 89% (*n* = 36), and 100% (*n* = 309) of AMs, AFs without dependent young, and AFs with dependent young, respectively (see also SWG 2016).

**Table 2:** Body condition scores (BCS) for polar bears in the Baffin Bay subpopulation 1993–1997 and 2011–2013

Bear	1990s			2000s		
	BCS = 1	BCS = 2	BCS = 3	BCS = 1	BCS = 2	BCS = 3
Adult female without dependent young	8	39	19	15	65	32
Adult female with 1 COY	14	12	7	12	40	2
Adult female with 2 COY	17	33	5	1	49	3
Adult female with 1 YRL	5	11	0	3	28	2
Adult female with 2 YRL	7	15	7	4	23	1
Subadult female	19	38	3	19	110	5
Subadult male	25	43	5	18	73	3
Adult male	56	143	127	73	232	47

*Notes*

BCS = 1 corresponds to a thin bear; BCS = 3 corresponds to a fat bear. Age classes are adult (≥5 yr), subadult (2–4 yr),yearling (YRL), and cub‐of‐the‐year (COY).

**Figure 4:**
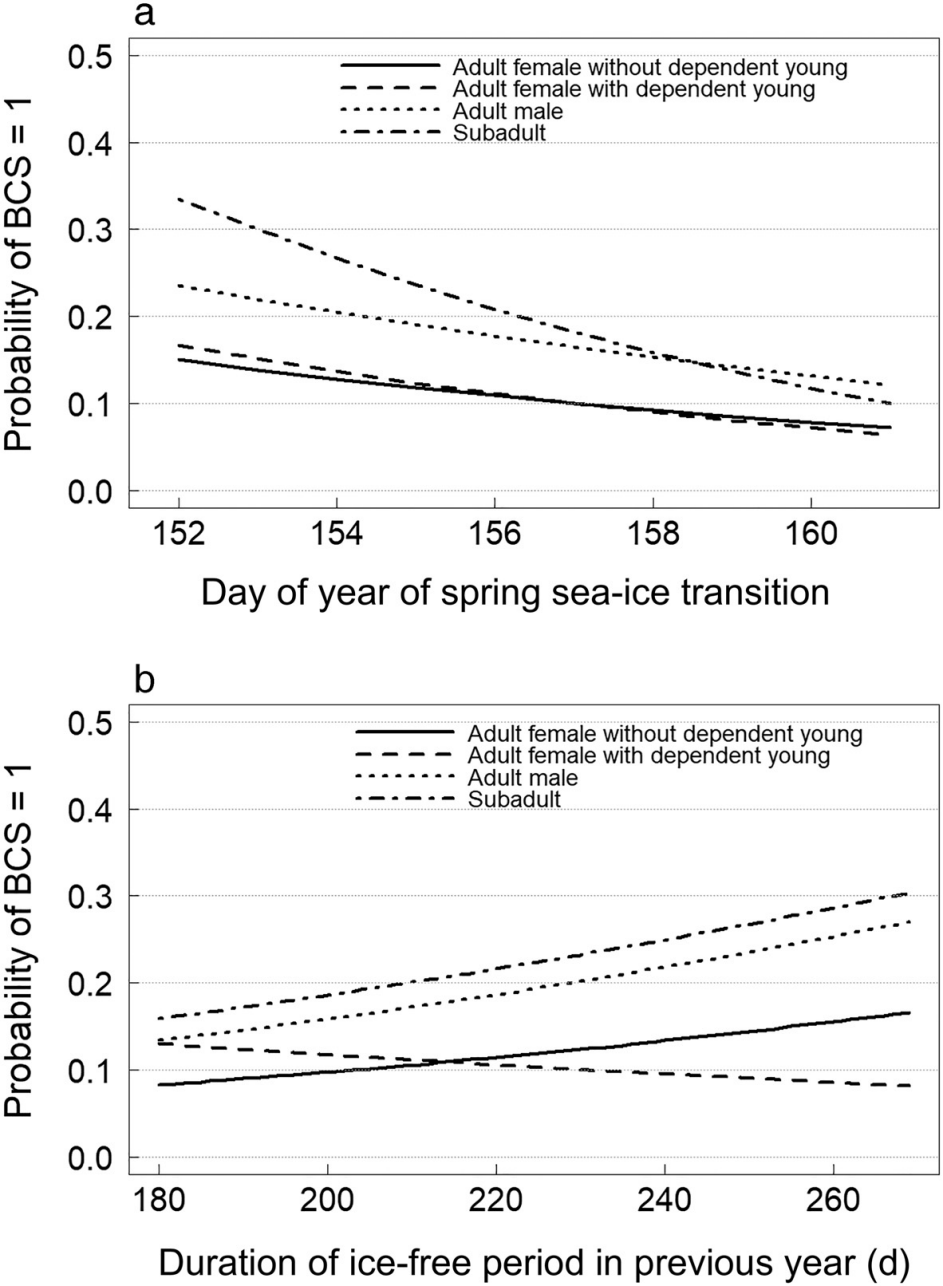
Relationships between sea ice conditions and the probability of body condition score (BCS) = 1 for the Baffin Bay polar bear subpopulation in the 2000s. Panel a demonstrates decreasing probabilities of BCS = 1 with later spring sea ice transition date (i.e., the covariate springtran_*t*_). Panel b demonstrates increasing probabilities of BCS = 1 with longer ice‐free periods in the preceding year (i.e., the covariate durfree_*t*−1_). Probabilities were estimated using the low‐AIC model for BCS with mean values for all continuous predictors other than the covariate that appears on the *x*‐axis.

There was no evidence for lack of fit in the general model for BCS (Hosmer and Lemeshow test, χ^*2*^ = 12.8, df* *=* *16, *P *=* *0.69). The low‐AIC model included 22 parameters indicating that BCS in year *t* varied as a function of sex, age, and reproductive class, spring sea ice transition date in year *t*, duration of the ice‐free period in the year *t* − 1, interactions between sea ice conditions and class, and an additive difference between the 1990s and 2000s (Table 3). The candidate model set included eight models with ΔAIC < 4 (Appendix S1: Table S1). Importance scores, defined as the sum of AIC weights for models in which a parameter appears, were 1.0 for springtran_*t*_, ts.springtran_*it*_, and durfree_*t*−1_, indicating strong support for relationships between sea ice and polar bear body condition. The sign and magnitude of estimated parameters generally supported a priori biological hypotheses. For example, under average sea ice conditions in the 2000s, AFs without dependent young had the highest probability of BCS = 3 (approximately 0.24), whereas AFs with dependent young, subadults, and AMs were 86%, 85%, and 50% less likely, respectively, to have BCS = 3. Similarly, AFs without dependent young had the lowest probability of BCS = 1 (approximately 0.11), whereas AFs with dependent young, subadults, and AMs were 2%, 91%, and 62% more likely, respectively, to have BCS = 1 (Fig. 4a,b). In general, increased sea ice availability increased the probability of BCS = 3, decreased the probability of BCS = 1, or both. BCS was more sensitive to changes in spring transition date for subadults compared to other classes (e.g., Fig. 4a), and less sensitive to changes in the duration of the ice‐free period in the previous year for AFs with dependent young compared to other classes (e.g., Fig. 4b).

**Table 3:** Results from the most‐supported multinomial logistic model for Body Condition Score (BCS) for the Baffin Bay polar bear subpopulation, 1993–1997 and 2011–2013. Model parameters are defined in the main text. Parameter estimates are provided with standard errors (SE), two‐sided *z*‐statistics, and *P*‐values resulting from Wald tests for significance. The reference value is BCS = 2

BCS Value		Intercept	*AFwC*_*it*_	*SUB*_*it*_	*AM*_*it*_	*p2000*_*t*_	*springtran*_*t*_	*ts.springtran*_*it*_	*durfree*_*t‐1*_	*AFwC*_*it*_ *× springtran*_*t*_	*AFwC*_*it*_ *× durfree*_*t‐1*_	*AM*_*it*_ *× springtran*_*t*_
BCS = 3	Estimate	−0.97	−20.74	−2.10	−16.27	−0.99	0.01	−0.02	0.00	0.12	−0.00	0.10
	SE	0.03	0.00	0.3	0.02	0.30	0.01	0.01	0.00	0.01	0.01	0.00
	*z*‐statistic	−33.77	−9343.25	−6.74	−1126.07	−3.29	2.92	−3.77	1.20	21.77	−0.39	79.52
	Wald *P*	0.00	0.00	0.00	0.00	0.00	0.00	0.00	0.23	0.00	0.70	0.00
BCS = 1	Estimate	12.19	7.19	0.30	10.03	−2.19	−0.09	−0.00	0.01	−0.03	−0.02	−0.06
	SE	0.02	0.00	0.27	0.01	0.22	0.01	0.01	0.00	0.01	0.01	0.00
	*z*‐statistic	531.44	1709.78	1.11	979.72	−9.85	−18.80	−0.34	3.65	−5.12	−3.43	−37.86
	Wald *P*	0.00	0.00	0.27	0.00	0.00	0.00	0.73	0.00	0.00	0.00	0.00

We performed a separate analysis of BCS for the 1990s data only, to determine whether the same relationships between BCS and sea ice were present during this 4‐yr time series, when data were collected by a single observer. Using the same procedures as the main analysis, the sea ice covariates springtran_*t*_, ts.springtran_*it*_, and durfree_*t*−1_ had importance scores of 0.95, 1.0, and 1.0, respectively. The sign and magnitude of sea ice effects were similar to the main analysis (i.e., more ice was associated with better body condition). This suggests that key ecological relationships were not driven by potential sampling differences between the 1990s and 2000s.

### Litter size and recruitment

During the 1990s, 88 AFs accompanied by 1–3 COY (144 COYs in total) and 45 AFs accompanied by 1–3 YRL (75 YRL in total) were sampled (Table 4). During the 2000s, 107 AFs accompanied by 1–2 COY (160 COYs in total) and 61 AFs accompanied by 1–2 YRL (89 YRL in total) were sampled.

**Table 4:** Mean litter size for cubs‐of‐the‐year (COY) and yearlings (YRL), and the annual number of litters observed (*n*), in the Baffin Bay polar bear subpopulation.

	COY		YRL	
Year	Litter size	*n*	Litter size	*n*
1993	1.65	17	1.71	7
1994	1.71	24	1.82	11
1995	1.50	22	1.64	14
1997	1.64	25	1.54	13
2011	1.48	33	1.57	21
2012	1.42	48	1.48	25
2013	1.65	26	1.27	15

*Note*

Litter sizes are conditional on the presence of at leastone cub (i.e., values do not reflect litter sizes of zero, for example in the case of whole‐litter loss).

The general model for COY litter size provided an adequate fit to the data (Hosmer and Lemeshow test, χ^2^ = 3.01, df* *=* *8, *P *=* *0.93). The low‐AIC model included five parameters indicating that COY litter size in year *t* varied as a function of spring sea ice transition date in year *t*, duration of the ice‐free period in the year *t* − 1, and maternal body condition at the time of sampling (Table 5). The candidate model set included 11 models with ΔAIC < 4 (Appendix S1: Table S2). Importance scores for BCS_*it*_, springtran_*t*_, and durfree_*t*−1_ were 1.0, 0.75, and 0.52, respectively, indicating strong support for relationships between maternal body condition and litter size, and moderate support for the additional influence of sea ice conditions in the current and previous year. The sign and magnitude of estimated parameters generally supported a priori biological hypotheses. For example, under average sea ice conditions in the 2000s, AFs with BCS = 2 had a higher probability of a two‐cub litter (approximately 0.24), whereas AFs with BCS = 1 were 53% less likely to have two cubs. AFs with BCS = 3 also were less likely to have two‐cub litters compared to adult females with BCS = 2 (Table 5). As expected, later spring sea ice transition dates were positively associated with having two‐cub litters, and longer ice‐free seasons in the previous year were negatively associated with having two‐cub litters (Table 5).

**Table 5:** Results from the most‐supported binomial logistic model for cub‐of‐the‐year (COY) litter size for the Baffin Bay polar bear subpopulation, 1993–1997 and 2011–2013. Model parameters are defined in the main text. Parameter estimates are provided with standard errors (SE), two‐sided *z*‐statistics, and *P*‐values resulting from Wald tests for significance

	Intercept	*springtran*_*t*_	*durfree*_*t‐1*_	*BCS*_*it*_ (BCS = 1)*	*BCS*_*it*_ (BCS = 3)*
Estimate	−10.17	0.07	−0.01	−1.24	−1.08
SE	4.81	0.03	0.00	0.40	0.56
*z*‐statistic	−2.12	2.51	−1.61	−3.09	−1.92
Wald *P*	0.03	0.01	0.11	0.00	0.05

* The reference value for the three‐level factor *BCS*_*it*_ is BCS = 2.

The general model for YRL litter size provided an adequate fit to the data (Hosmer and Lemeshow test, χ^2^ = 6.02, df* *=* *8, *P *=* *0.65). The low‐AIC model included two parameters, indicating that YRL litter size differed between the 1990s and 2000s (β_4_ *= *−0.759, SE = 0.404. *P *=* *0.06). The candidate model set included 23 models with ΔAIC < 4, reflecting a high degree of model‐selection uncertainty. Importance score was the highest (0.69) for the parameter p2000_*t*_, indicating moderate support for a change in YRL litter size across decades. There was relatively low support for the effects of sea ice conditions or maternal body condition on YRL litter size. Based on the low‐AIC model, the probability that an adult female had a two‐cub litter in the 1990s (approximately 0.64) was 29% higher than in the 2000s.

The mean number of COY and YRL per AF varied across years (Table 6). The number of COY per AF was higher in the 1990s (mean = 0.72, standard error of the mean [SEM] = 0.06) than in the 2000s (mean = 0.57, SEM = 0.05; *P *=* *0.03). The number of YRL per AF in the 1990s (mean = 0.38, SEM = 0.05) was not statistically different from the 2000s (mean = 0.32, SEM = 0.04) at a significance level of 0.05 (*P *=* *0.18). For the four instances that annual sampling occurred in two successive years, mean COY per AF in year *t* was positively associated with mean YRL per AF in year *t *+* *1 (Kendall's tau = 1.0, *P *<* *0.01), which was likely a function of cohort effects with higher COY production subsequently resulting in higher YRL recruitment.

**Table 6:** Mean numbers of cubs‐of‐the‐year (COY) and yearlings (YRL) per adult female (AF) for the Baffin Bay polar bear subpopulation, 1993–1997 and 2011–2013

Year	COY per AF	YRL per AF
1993	0.80 (0.07)	0.34 (0.11)
1994	0.82 (0.16)	0.39 (0.04)
1995	0.69 (0.10)	0.48 (0.07)
1997	0.63 (0.16)	0.31 (0.03)
2011	0.60 (0.10)	0.41 (0.08)
2012	0.55 (0.06)	0.30 (0.02)
2013	0.57 (0.06)	0.25 (0.04)

*Note*

Values in parentheses represent standard errors of the mean (SEM).

### Projecting reproductive metrics

**Figure 5:**
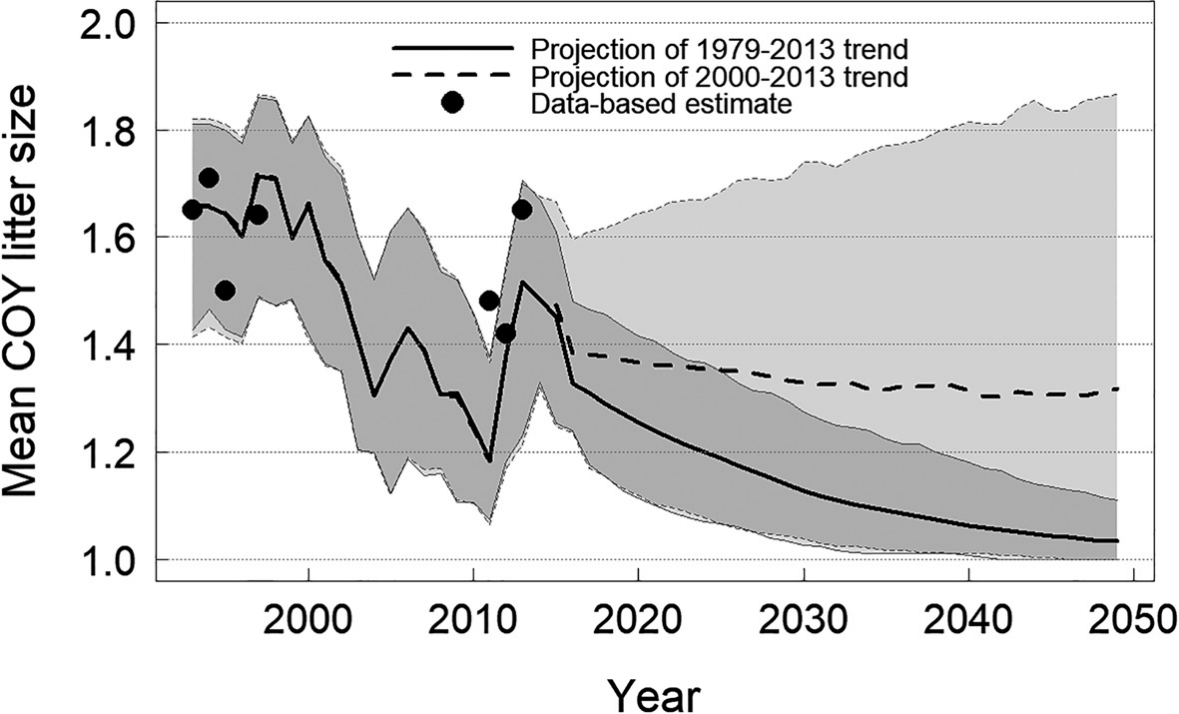
Example projections of mean cub‐of‐the‐year (COY) litter size for the Baffin Bay polar bear subpopulation if the linear trend in sea ice loss observed since 1979 continued. Projections are based on the most‐supported models for adult female BCS and COY litter size. The solid line represents COY litter size as a function of linear projections of sea ice metrics for the period 1979–2013, during which sea ice declined significantly. The dashed line represents COY litter size as a function of linear projections of sea ice metrics for the period 2000–2013, during which sea ice was relatively stable for comparison. The black circles are data‐based estimates of mean litter size.

Analyses of sea ice metrics, body condition, and reproduction were linked together to project COY litter size three polar bear generations into the future (Fig. 5). For the purpose of sensitivity analysis, we projected litter size using linear projections of sea ice metrics based on (1) data for the period 1979–2013, during which sea ice declined (slope[springtran_*t*_] = −0.83 d/yr, SE = 0.13, *P *<* *0.001; slope[durfree_*t*−1_] = 1.85 d/yr, SE = 0.36, *P *<* *0.001), and (2) data for the period 2000–2013, during which sea ice was largely stable (slope[springtran_*t*_] = −0.21 d/yr, SE = 0.49, *P *=* *0.67; slope[durfree_*t*−1_] = 0.29 d/yr, SE = 1.54, *P *=* *0.85). Projections demonstrate that the reproductive success of BB polar bears is sensitive to sea ice conditions and can be expected to decline in coming decades if ice loss continues and the estimated relationships between sea ice, body condition, and litter size do not change.

## Discussion

We analyzed multiple types of data on habitat use, body condition (i.e., fatness), and cub production during a period of rapid habitat loss for the BB polar bear subpopulation. Our analyses indicate that reduced sea ice availability has been associated with longer times spent on land, reduced body condition, and reduced reproductive success. To our knowledge, this is the first large‐scale analysis for polar bears using data from both visual observation and physical capture to evaluate long‐term patterns of ecological change. Consistency between our findings and smaller‐scale physical capture studies (Rode et al. 2012, 2014, Bromaghin et al. 2015, Lunn et al. 2016) suggests that biopsy darting and aerial observation, when performed by experienced observers following a consistent protocol, can provide information on subpopulation status relevant to management and conservation. This information can be obtained at a lower cost and with less stress to animals compared to physical capture. Collectively, our findings provide an improved understanding of the temporal and functional relationships characterizing the effects of habitat loss on an apex predator.

### Increased period on land and association with sea ice retreat

The coast of Baffin Island is generally the last place in Baffin Bay to lose sea ice in summer, and the first place in Baffin Bay to regain sea ice in fall. This dynamic appears to have structured the overall movements of the BB subpopulation, in that most bears follow the sea ice as it recedes towards Baffin Island in late spring and summer, presumably to maximize on‐ice foraging opportunities, and thus end up on Baffin Island for the ice‐free season (Ferguson et al. 1997, 2000a, Taylor et al. 2001). General assessments of sea ice habitat demonstrate that the Baffin Bay region has experienced a long‐term reduction in sea ice cover and a trend towards earlier spring breakup and later fall freeze‐up (Stirling and Parkinson 2006, Stern and Laidre 2016), consistent with declining sea ice trends across the Arctic (Stroeve et al. 2012, Parkinson 2014). Experienced polar bear hunters living on the coasts of Baffin Bay have reported changing sea ice conditions during the last few decades and noted effects on the distribution and body condition of polar bears (Dowsley and Wenzel 2008, Born et al. 2011).

The sea ice metrics used in our analyses were designed to mark the annual transition from winter to summer sea ice conditions (date of retreat) and from summer to winter sea ice conditions (date of advance), and not necessarily to predict the behavior of polar bears (Stern and Laidre 2016). Nevertheless, temporal trends were similar for the sea ice metrics and the dates of polar bear arrival on land and departure from land, indicating that ice dynamics are useful predictors of land use in the BB region. The offsets of the trend lines in Fig. 3 suggest that the 50% ice coverage threshold used to define transition dates was conservative, in the sense that polar bears remained on the sea ice in spring well past our date of sea ice retreat, and returned to the sea ice in fall before our date of sea ice advance. Depending on the objectives of a particular analysis, the threshold could be modified (e.g., set to a lower value that would provide an improved match between date of retreat and arrival on land, and between date of advance and departure from land). Different thresholds could be evaluated for different polar bear management regions (Stern and Laidre 2016).

We found that polar bears in Baffin Bay spent approximately one month longer on land in the 2000s than in the 1990s. This is similar to changes in land use for other seasonal sea ice subpopulations (Rode et al. 2015, Atwood et al. 2016, Lunn et al. 2016). Adult females in Baffin Bay in the 2000s no longer spend the summer on Devon or Ellesmere islands due to early ice breakup and lack of sea ice adjacent to these areas, but only use Baffin Island on the Canadian side of Baffin Bay. While our use of 5‐km buffer to identify land use might have resulted in some offshore bears being classified as on land, this was unlikely during the focal spring and fall time periods because landfast ice was either not present or newly forming. This 5‐km buffer also follows that used in analyses in other regions (Atwood et al. 2016).

The arrival and departure dates in both decades were closely related to sea ice metrics representing spring and fall transition dates. Several studies for polar bears have identified relationships between these metrics and vital rates (Regehr et al. 2007, 2010, Lunn et al. 2016, Laidre et al. 2018a, b), while other studies have used similar metrics as proxies for environmental carrying capacity during population projections (Regehr et al. 2016). Although Stern and Laidre (2016) proposed that these metrics capture important spatial and temporal dynamics of polar bear habitat in a biologically meaningful way, this is the first study to evaluate interrelationships among environmental conditions, habitat use, body condition, and demographic effects.

We found that the ice‐free period in Baffin Bay, during which ice seals are not accessible and polar bears are reliant on stored energy reserves, has increased from about 60 d in the 1990s to over 90 d in the 2000s. Bears coming off the sea ice in summer are thought to be nearing their annual peak in body condition due to the spring period of hyperphagia when juvenile ringed seals are relatively abundant and susceptible to predation (Smith 1980, Stirling and Øritsland 1995). Studies of maximum fasting duration in polar bears suggest that, at 180 d, 56–63% of subadults and 18–24% of adult males could die of starvation (Molnár et al. 2010, 2014, Pilfold et al. 2016), establishing the limits of bears to withstand lengthening ice‐free seasons. Recent capture–recapture analyses for the BB subpopulation did not find evidence for relationships between sea ice availability and survival (SWG 2016). Such relationships also have not been identified for some polar bear subpopulations that are experiencing sea ice loss but inhabit biologically productive regions (e.g., the Chukchi Sea; Regehr et al. 2018). However, it is not clear whether this finding provides evidence that the current 90‐d ice‐free season in BB is sufficient to maintain survival, despite the apparent negative effects on BCS and reproductive indices. Other studies suggest a lag of multiple years between detection of declining body condition and declining demographic status (Stirling et al. 1999, Regehr et al. 2007). Furthermore, SWG (2016) indicated that small sample sizes, high variability in environmental conditions, and nonrandom temporary emigration for the BB subpopulation likely compromised the ability to evaluate potential relationships between survival and environmental conditions.

### Melville Bay resident bears

In this study, we documented, for the first time, habitat use patterns of polar bears that remain along the Northwest Greenland coast during the open‐water season. Traditional knowledge from polar bear hunters in Northwest Greenland (Born et al. 2011) and opportunistic observations (e.g., Taylor et al. 2001) previously suggested that some bears use this strategy. Bears in our study remained in Northwest Greenland year‐round, in some cases spending >2.5 consecutive years on fast ice at the base of glacier fronts, and on the glaciers or glacial mélange during summer. It is not possible to use satellite telemetry data to compare whether the frequency of glacier‐front use in West Greenland has increased because only one satellite collar was deployed at glacier fronts in the 1990s (Taylor et al. 2001) despite the area being thoroughly searched (Born et al. 1992, Rosing‐Asvid 1993). However, field observations suggest there is currently more activity by bears on the fast ice in Melville Bay and at glacier fronts; in the 1990s only ~8% (2 of 24) of captures of independent polar bears (i.e., adults and subadults) occurred close to land and at glacier fronts (Born et al. 1992, Rosing‐Asvid 1993), whereas in the 2000s, 25% of captures occurred in this area (SWG 2016). A potential increase in spring polar bear densities in Melville Bay would be consistent with information from experienced polar bear hunters (Born et al. 2011). In both the 1990s and 2000s surveys, ringed seals appeared abundant in Melville Bay (Born et al. 1999; E. W. Born and K. L. Laidre, *unpublished data*). The area was established as a sanctuary (Melville Bay Nature Reserve) in 1980, which protects nearshore areas from hunting (Born et al. 2011). Similar habitat use patterns have been observed in the Barents Sea, where some females remain in fjords of Svalbard over many years while others range over vast areas of the Barents Sea (Wiig 1995, Mauritzen et al. 2001, 2002, Aars et al. 2017). Further, Freitas et al. (2013) demonstrated the importance of fast‐ice areas for polar bears in Svalbard, particularly the biologically productive glacier fronts, especially for females with COYs. It is possible that the year‐round habitat provided by glacier fronts will become increasingly important to polar bears and other pagophilic species as loss of sea ice continues. This may fragment bears into smaller isolated subpopulations with highly specific adaptations (e.g., use of glaciers).

### Body condition

Declining body condition and reproduction are amongst the first subpopulation‐level effects to occur in polar bears as a result of habitat loss due to climate change (Stirling and Parkinson 2006, Molnár et al. 2011, Stirling and Derocher 2012). Our evidence of a decline in condition in the BB subpopulation from 1993 to 2013, along with similar findings from a previous study (Rode et al. 2012), is accompanied by evidence of concurrent reproductive declines. The finding that annual variation in the body condition of adult bears is associated with spring sea ice transition date is consistent with the hypothesis that reduced access to prey during the important spring to early summer feeding period, either due to less time on the ice or increased intraspecific competition while on the ice, is likely one of the mechanisms driving this decline. Our findings also are consistent with local and traditional knowledge suggesting that body condition of polar bears in Baffin Bay was poorer in the early 2000s relative to the 1990s (Dowsley and Wenzel 2008, Born et al. 2011). A genetic mark–recapture study estimated a mean subpopulation size of 2,826 (95% CI = 2,059–3,593) during the period 2012–2013 (SWG 2016), suggesting that BB remains among the largest polar bear subpopulations (PBSG 2018). However, that analysis was not able to determine trends in abundance. The finding that changes in BCS and reproduction were associated with sea ice availability suggests that, now or in the near future, sea ice loss due to climate warming may result in a declining environmental carrying capacity for the BB subpopulation.

Using quantitative body condition metrics derived from morphometric measurements of captured polar bears, Rode et al. (2012) detected a decline in the condition of BB polar bears between 1990 and 2010 concurrent with declining sea ice cover. Our results extend those findings and suggest that trends in body condition and the association with sea ice conditions have continued beyond 2010. Furthermore, our results demonstrate that body condition was a function of sex, age, and reproductive class: AFs without dependent young generally had the highest body condition, followed by AMs, AFs with dependent young, and finally subadults.

Body condition for BB polar bears was strongly related to spring sea ice transition date in the same year, days elapsed between breakup and the time of observation (i.e., bears became leaner with longer time spent on land), and duration of the ice‐free season in the previous year (except for AFs with dependent young) suggesting a multiyear lag in the effects of environmental variation (Table 3). Body condition for subadults was most sensitive to spring transition date (Fig. 4a), consistent with the lower energy reserves and increased metabolic demands of structural growth in younger bears and consistent with the finding that survival of subadults seems to be more sensitive to sea ice conditions than other age classes (Regehr et al. 2007, Lunn et al. 2016). The mean probability of AFs with dependent young having BCS = 1 exhibited a slight negative relationship with the duration of the ice‐free period in the preceding year (i.e., a longer ice‐free period was associated with a lower probability of BCS = 1; Fig. 4b). This negative relationship differed from other sex, age, and reproductive classes but was not significant (e.g., considering statistical uncertainty there was a 0.14 probability that the relationship was positive), resulting in ambiguous evidence regarding the relationship between body condition of AFs with dependent young and the duration of the ice‐free period in the previous year. This may be due in part to the fact that most AFs with COY emerge from the maternity den in poor condition due to the extended fasting period associated with reproduction (i.e., up to eight months), which means that their condition during autumn sampling was mostly a function of environmental conditions since den emergence (i.e., spring transition date in the current year).

The BCS used in our study is a qualitative, categorical index that is a coarser and potentially less precise measure of condition than the metrics derived from physical capture data by Rode et al. (2012). Thus, our BCS data may be less sensitive to changes in body condition over time or in response to ecological parameters (Vongraven et al. 2012, McKinney et al. 2014). Additional variance may have been introduced in the body condition data during the latter years of our study (2011–2013), when condition was assessed based on examination from a distance rather than physical handling. Several lines of evidence suggest observer bias was not an important factor in our study. Differences between observers are unlikely to explain the close association between BCS and sea ice conditions, which were highly variable from year to year. As noted by Stirling et al. (2008), the FI from which our condition metric was derived has been found to be consistent among different observers when blind comparisons are conducted. In other studies, FI data collected by multiple observers have been found to correlate closely with quantitative indices of condition (e.g., Stirling et al. 2008, McKinney et al. 2014). Therefore, while we cannot exclude the possibility of observer bias, this potential source of bias is unlikely to account for our observed patterns.

Measures of body condition have been identified as a key metric of polar bear health (Patyk et al. 2015). While direct measurement of body mass or body condition by morphometry or adipose tissue lipid content (McKinney et al. 2014) remain the most accurate means of monitoring condition, our findings suggest that long‐term trends in body condition can be detected using FI scores acquired without the physical immobilization of animals. Similar to previous studies (e.g., Stirling et al. 2008, McKinney et al. 2014), we have demonstrated the utility of a simple qualitative metric for monitoring trends in body condition of polar bears. In circumstances where demographic studies are conducted periodically or where survey methods do not involve capture and handling, visually assigned body condition scores from research surveys, harvested bears, community‐based surveys, or opportunistic observation may represent a viable monitoring tool.

### Litter size and recruitment

We found that COY litter size was a function of the mother's body condition, spring transition date, and, to a lesser degree, duration of the ice‐free period in the previous year. This suggests that, for AFs with COY that did not experience whole‐litter loss, the mother's body condition in autumn, which likely integrates environmental conditions and hunting success since den emergence, was a primary determinant of COY survival. The fact that spring sea ice transition date also was a supported predictor suggests the presence of environmental effects on COY survival beyond those that were reflected in our three‐level BCS score for maternal body condition. The influence of sea ice conditions in the previous year on COY litter size is consistent with other studies suggesting that maternal body condition prior to parturition is positively correlated with both mass and survival of new cubs (Atkinson and Ramsay 1995). Similar declines in reproduction over time, and in association with sea ice conditions, have been reported for other polar bear subpopulations (Derocher and Stirling 1995, Derocher 2005, Rode et al. 2010, 2014, Peacock et al. 2013). Interestingly AFs with BCS = 3 were less likely to have two‐cub litters compared to adult females with BCS = 2. This may reflect the two‐way relationship between maternal condition and litter size: the high energetic cost of supporting two cubs makes it difficult for adult females to achieve BCS = 3, even under favorable environmental conditions.

YRL litter size was not associated with maternal body condition at the time of observation or with sea ice conditions, although our analyses indicated that YRL litter size likely declined between the 1990s and 2000s. We are not aware of other potential explanations for this finding other than loss of sea ice habitat. YRL survival may be a more complicated function of maternal body condition and environmental variation, at multiple temporal scales, that was beyond our ability to evaluate in the current analysis.

In this study, the number of COY per AF was significantly lower in the 2000s than the 1990s. This suggests that COY production has declined over time, likely due to a combination of reduction in breeding probability (i.e., the probability that an AF available to breed in year *t* produced a litter of COY in year *t* + 1), which could not be directly estimated in the current study; and reductions in COY survival, as evidenced by declining COY litter size.

YRL recruitment has been identified as an important reproductive metric in polar bear subpopulations because it integrates both natality and COY survival (Vongraven et al. 2012). The expected number of YRL per AF declined across decades although this was not statistically significant. It is unclear if the lack of a significant decline was related to lower power to detect changes in YRL recruitment due to smaller sample size, the fact that most mortality of young polar bears is experienced by COYs during the first 8 months of life, or the existence of a compensatory mechanism such that individual COY survival increases when litter size is reduced. Estimated values of YRL per AF between 1993 and 2013 ranged from 0.26 to 0.48, suggesting that recruitment is sufficient for the BB subpopulation to be viable, given adequate survival rates (Regehr et al. 2017b). Additional insight into reproductive performance and viability might be gained from an analysis of mark–recapture data using a multistate model based on the life history of polar bears (e.g., Regehr et al. 2018) coupled with a population viability analysis based on the estimated vital rates and their relationships with environmental conditions.

### Projections of reproductive performance with projected sea ice loss

We demonstrated how relationships between environmental conditions, body condition, and litter size might be used to project future reproductive performance. To our knowledge, such projections have not been made based on empirical data for polar bears outside of multistate capture–recapture modeling (e.g., Regehr et al. 2010, 2018, Lunn et al. 2016), which has not been feasible for most subpopulation assessments. Furthermore, estimates of sea ice availability and individual BCS based on large‐scale visual observation have not previously been related to reproductive performance. Our findings are consistent with other studies showing a positive relationship between maternal body size or condition and cub survival (Folio et al. 2019), suggesting that with sufficient sample size it is possible to inform projections of demographic status for polar bears based on visual assessment of BCS together with data on sex, age, and reproductive composition. Projection of COY litter size based on linear projections of sea ice conditions during the period 1979–2013 (Fig. 5, solid line) suggests the potential for reproductive declines if sea ice loss continues. Such declines are conceptually similar to linear projections of a proxy for environmental carrying capacity used in a harvest risk assessment for the BB subpopulation (Regehr et al. 2017b) and a recent Red List assessment for the global population of polar bears (Wiig et al. 2015, Regehr et al. 2016). The scope of current analyses, however, did not include assessment of the assumptions inherent in such projections, which include that relationships between sea ice conditions, BCS, and litter size will remain stationary beyond the range of observed conditions. Therefore, we also projected litter size as a function of sea ice conditions with significant inter‐annual variation but no significant temporal trend (Fig. 5, dashed line), to demonstrate the sensitivity of projected outcomes to future environmental conditions. Our projections of mean COY litter size may not reflect the overall productivity of the BB subpopulation because additional factors such as breeding probability, whole‐litter‐loss, and yearling mortality are not included. Similar litter sizes for COY and yearlings, together with the smaller number of yearling observations, suggest that survival of individual COY litter mates from the autumn of year *t* to year *t *+* *1 is not independent (i.e., that litters are kept or lost as a unit).

## Conclusion

The impact of climate change on the world's biota has manifested broadly, resulting in latitudinal range shifts, advancing dates of arrival of migrants and onset of breeding, and altered ecosystems (Post et al. 2009, 2019). For ice‐dependent species in polar ecosystems, climate change has resulted in heightened conservation concerns because of negative impacts of sea ice loss on most aspects of these species' life history (e.g., Derocher et al. 2013, Reimer et al. 2019). Over the next century, the conservation of polar bears will depend not only on our ability to understand and quantify the effects of climate change, but also on our capacity to predict how climate change will influence viability and adjust management actions accordingly (Regehr et al. 2017a).

Our analyses indicate that reduced sea ice availability has been associated with longer times spent on land, reduced body condition, and reduced reproductive success in Baffin Bay polar bears. This subpopulation inhabits a seasonal ice ecoregion (Amstrup et al. 2008), which is characterized by the complete melting of sea ice in summer that forces bears onshore for an extended period of time while they wait for the sea ice to reform in the fall. Although this general pattern has not changed over time, the spring sea ice retreat now occurs earlier and the fall advance later (Stern and Laidre 2016). Bears are coming ashore earlier in the summer, departing offshore later, and spending up to 30 additional days onshore in the 2000s than in the 1990s. Concurrently, body condition and reproductive performance have declined. We documented strong relationships between sea ice conditions and declines in body condition and COY litter size. Our findings are consistent with the predicted effects of climate change on polar bears (Molnár et al. 2010, 2014, Stirling and Derocher 2012), the empirical evidence available for some subpopulations (e.g., Regehr et al. 2007, Rode et al. 2010, 2012, Lunn et al. 2016, Obbard et al. 2016), and with the assumption of a declining environmental carrying capacity for the BB subpopulation in a recent harvest risk assessment (Regehr et al. 2017b). We note, however, that the functional and temporal relationships between declines in body condition and recruitment, and declines in subpopulation size, are poorly understood and that the trend of the BB subpopulation is currently unknown (SWG 2016). Although polar bears still occupy much of their historic range (PBSG 2018), the unidirectional nature of sea ice loss has raised long‐term conservation concerns for this species and other Arctic marine mammals (Stirling and Derocher 2012, Derocher et al. 2013, Laidre et al. 2015a).

## Data Availability

Data are available from the Dryad Digital Repository: http://doi.org/10.5061/dryad.ht76hdrbb
