# Supplementary material for: Interrelated ecological impacts of climate change on an apex predator
Source: Ecol Appl. 2020 Feb 4;30(4):e02071. doi: 10.1002/eap.2071 (PMC7317597; doi:10.1002/eap.2071)
Supplement: Supplementary file 1 [file EAP-30-e02071-s001.pdf]

Supporting Information. Laidre, K. L., S. Atkinson. E. V. Regehr, H. L. Stern, E. W. Born, Ø. Wiig, N. J. Lunn, and M. Dyck. 2020. Interrelated ecological impacts of climate change on an apex predator. Ecological Applications.

Appendix S1

TABLE S1. Model selection table for multinomial logistic models of Body Condition Score (BCS) for the Baffin Bay polar bear subpopulation, 1993-1997 and 2011-2013. Model parameters are defined in the main text. Models M2,M3,… M8 are models with Akaike's Information Criteria (AIC) values within 4 of the most-supported model M1. For each model we report the associated degrees of freedom (df), log-likelihood (logLik), AIC, change in AIC with respect to the most-supported model (ΔAIC), and AIC weight (w). For each parameter, Importance is the sum of AIC weights for candidate models in which the parameter appears.

Parameter	M1	M2	M3	M4	M5	M6	M7	M8	Importance
Intercept	+	+	+	+	+	+	+	+	NA
$AFwC_{it}$	+	+	+	+	+	+	+	+	1.0
SUB_{it}	+	+	+	+	+	+	+	+	1.0
AM_{it}	+	+	+	+	+	+	+	+	1.0
$p2000_t$	+	+	+	+	+	+	+	+	1.0
$springtran_t$	+	+	+	+	+	+	+	+	1.0
$ts.springtran_{it}$	+	+	+	+	+	+	+	+	1.0
$durfree_{t-1}$	+	+	+	+	+	+	+	+	1.0
$AFwC_{it} \times springtran_t$	+		+	+	+				0.6
$AFwC_{it} \times durfree_{t-1}$	+	+	+	+	+	+	+	+	1.0
$SUB_{it} \times springtran_t$			+			+			0.2
$SUB_{it} \times durfree_{t-1}$				+				+	0.1
$AM_{it} \times springtran_t$	+	+	+	+	+	+	+	+	1.0
$AM_{it} \times durfree_{t-1}$					+		+		0.1
df	22	20	24	24	24	22	22	22	
logLik	-1252.4	-1254.9	-1251.2	-1251.7	-1251.8	-1253.9	-1254.0	-1254.0	
AIC	2548.7	2549.7	2550.4	2551.4	2551.5	2551.8	2551.9	2552.1	
ΔAIC	0.00	0.98	1.65	2.63	2.77	3.01	3.17	3.32	
weight	0.31	0.19	0.14	0.08	0.08	0.07	0.06	0.06	

TABLE S2. Model selection table for binomial logistic models of cub-of-the-year (COY) litter size for the Baffin Bay polar bear subpopulation, 1993-1997 and 2011-2013. Model parameters are defined in the main text. Models M2, M3,… M11 are models with Akaike's Information Criteria (AIC) values within 4 of the most-supported model M1. For each model we report the associated degrees of freedom (df), log-likelihood (logLik), AIC, change in AIC with respect to the most-supported model (ΔAIC), and AIC weight (w). For each parameter, Importance is the sum of AIC weights for candidate models in which the parameter appears.

Parameter	M1	M2	M3	M4	M5	M6	M7	M8	M9	M10	M11	Importance
Intercept	+	+	+	+	+	+	+	+	+	+	+	NA
$springtran_t$	+		+	+	+	+	+			+	+	0.750
$ts.springtran_{it}$					+		+	+		+	+	0.310
$durfree_{t-1}$	+		+		+		+		+			0.520
$p2000_t$		+	+		+	+		+	+		+	0.610
BCS_{it}	+	+	+	+	+	+	+	+	+	+	+	1.000
df	5	4	6	4	7	5	6	5	5	5	6	
logLik	-125.682	-126.776	-124.820	-126.995	-124.126	-126.366	-125.581	-126.718	-126.775	-126.797	-126.302	
AIC	261.400	261.600	261.600	262.000	262.300	262.700	263.200	263.400	263.600	263.600	264.600	
ΔAIC	0.00	0.19	0.27	0.63	0.89	1.37	1.80	2.07	2.19	2.23	3.24	
weight	0.16	0.15	0.14	0.12	0.10	0.08	0.07	0.06	0.05	0.05	0.03	
